# A High Quality Genome for *Mus spicilegus*, a Close Relative of House Mice with Unique Social and Ecological Adaptations

**DOI:** 10.1534/g3.118.200318

**Published:** 2018-05-24

**Authors:** Matthew B. Couger, Lena Arévalo, Polly Campbell

**Affiliations:** * High Performance Computing Center; † Department of Integrative Biology, Oklahoma State University, Stillwater, OK 74078

**Keywords:** *de novo* genome assembly, mound-building mouse, *Mus spicilegus*, read cloud, vomeronasal receptors

## Abstract

Genomic data for the closest relatives of house mice (*Mus musculus* species complex) are surprisingly limited. Here, we present the first complete genome for a behaviorally and ecologically unique member of the sister clade to house mice, the mound-building mouse, *Mus spicilegus*. Using read cloud sequencing and *de novo* assembly we produced a 2.50 Gbp genome with a scaffold N50 of 2.27 Mbp. We constructed >25 000 gene models, of which the majority had high homology to other *Mus* species. To evaluate the utility of the *M. spicilegus* genome for behavioral and ecological genomics, we extracted 196 vomeronasal receptor (VR) sequences from our genome and analyzed phylogenetic relationships between *M. spicilegus* VRs and orthologs from *M. musculus* and the Algerian mouse, *M. spretus*. While most *M. spicilegus* VRs clustered with orthologs in *M. musculus* and *M. spretus*, 10 VRs with evidence of rapid divergence in *M. spicilegus* are strong candidate modulators of species-specific chemical communication. A high quality assembly and genome for *M. spicilegus* will help to resolve discordant ancestry patterns in house mouse genomes, and will provide an essential foundation for genetic dissection of phenotypes that distinguish commensal from non-commensal species, and the social and ecological characteristics that make *M. spicilegus* unique.

As the premier mammalian model system in biomedical, evolutionary, and quantitative genetics, the genomic and bioinformatic resources for house mice (*Mus musculus* species complex) are unparalleled in mammals (Mouse Genome Sequencing Consortium *et al.* 2002; Valdar *et al.* 2006; Keane *et al.* 2011; Nicod *et al.* 2016; Morgan *et al.* 2016; Eppig *et al.* 2017). In contrast, genomic resources for the closest relatives of house mice are surprisingly limited, and this constrains the evolutionary scope of comparative and experimental studies. House mice are sister to a clade of Eurasian, non-commensal (aboriginal) species that includes *M. spicilegus*, *M. macedonicus*, and *M. cypriacus* (Suzuki *et al.* 2004; 2013; Macholán *et al.* 2012). Whereas phylogenies based on a small number of loci have placed the Algerian mouse, *M. spretus*, as basal to both clades (Macholán *et al.* 2012; Suzuki *et al.* 2013), recent analysis using whole exome sequences place *M. spretus* as the basal member of the clade containing *M. spicilegus* (Sarver *et al.* 2017).

The availability of a medium coverage (20x) genome for *M. spretus* (Keane *et al.* 2011) has provided key insight into genome structure and molecular evolution in house mice (*e.g.*, Nellåker *et al.* 2012; Wynn *et al.* 2012; Baker *et al.* 2015). However, *M. spretus* has a history of introgression with house mouse subspecies, *M. m. domesticus* (Song *et al.* 2011; Liu *et al.* 2015), and ∼12% of loci in *M. musculus* subspecies genomes place *M. spretus* within the house mouse clade (Keane *et al.* 2011). Both of these factors may complicate analyses using only *M. spretus* as a close outgroup to house mice. Moreover, complete genome sequences for additional Eurasian *Mus* species will enable genetic dissection of the ecological and behavioral adaptations that differentiate aboriginal from commensal species. The power of genomic data for a suite of ecologically diverse congeners is illustrated by the Drosophila 12 genomes project (Drosophila 12 Genomes Consortium *et al.* 2007), and the wide applications of these resources (*e.g.*, Haerty *et al.* 2007; Croset *et al.* 2010; Nourmohammad *et al.* 2017).

Here, we present the first complete genome sequence for the mound-building mouse, *M. spicilegus*. This Eastern European species occurs from the Austro-Hungarian border, east to the Ukraine and south to the Black Sea, with disjunct distribution in Montenegro, Albania, and Greece (Coroiu *et al.* 2016) (Figure 1). We chose *M. spicilegus* for three main reasons. First, given historic introgression and moderate phylogenetic discordance between *M. musculus* lineages and *M. spretus*, the inclusion of another close relative of house mice is desirable for resolution of ancestry patterns across house mouse genomes (*e.g.*, Keane *et al.* 2011). Second, both *M. spicilegus* and *M. spretus* exhibit behaviors consistent with social monogamy, including paternal care (Patris and Baudoin 2000; Cassaing *et al.* 2010). Yet male reproductive phenotypes suggest that the opportunity for sperm competition in both species is significantly higher than in house mice (Gómez Montoto *et al.* 2011), in which female multiple mating is common (Dean *et al.* 2006; Thonhauser *et al.* 2010). Complete genome sequences for both *M. spicilegus* and *M. spretus* will facilitate work on the genetic basis of these intriguing observations. Third, behaviors and life-history traits associated with over-winter survival in *M. spicilegus* are unique among Eurasian *Mus* and completely unstudied at the genetic level.

**Figure 1:**
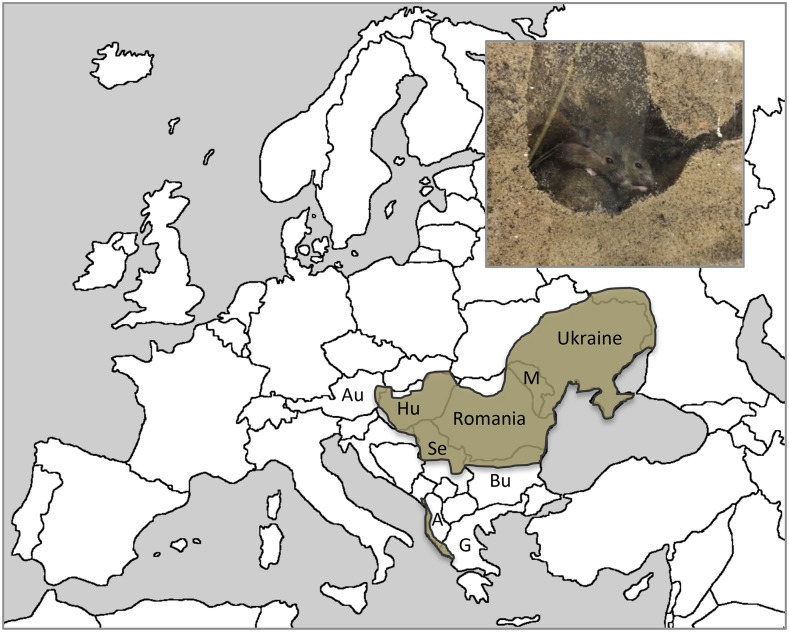
The geographic distribution of the mound-building mouse, *Mus spicilegus*. Inset: Mound-building mice are highly social and exhibit natural burrowing behavior under laboratory conditions. Au, Austria; Hu, Hungary; Se, Serbia; Bu, Bulgaria; M, Moldova; A, Albania; G, Greece. Distribution based on Coroiu *et al.* (2016). Photo, AG Ophir.

As the species’ common name suggests, *M. spicilegus* builds large mounds of soil and vegetation that serve a thermoregulatory function (Szenczi *et al.* 2011; 2012). Mounds, and the complex burrow systems they protect, are constructed in the autumn by young of the year that spend the winter underground and delay reproduction until the following spring (Garza *et al.* 1997; Poteaux *et al.* 2008; Szenczi *et al.* 2011). Mound and burrow construction takes several days to weeks and is thought to involve division of labor (Hurtado *et al.* 2013), a key feature of cooperative behaviors (Beshers and Fewell 2001). Higher within *vs.* between mound relatedness based on eight microsatellites suggest that cooperative mound construction is favored by kin selection (Garza *et al.* 1997). The availability of a genome for *M. spicilegus* will facilitate larger scale analyses of relatedness and population structure, and exploration of the genetic basis of behaviors unique to this species.

## Materials And Methods

### Strain selection, library creation and sequencing

We sequenced the genome of a male *M. spicilegus* from the wild-derived strain, ZRU. The strain was developed at the Wild Mouse Genetic Repository (Montpellier, France) using wild-caught founders from Kalomoyevka, Ukraine, captured in 1982. Very high molecular weight DNA was isolated from liver tissue using pulse field electrophoresis for 10x Genomics high throughput linked read sequencing. Libraries were prepared using the Chromium Genome v2 Library Kit and were barcoded for linking using the 10x Chromium microfluidic platform. Chromium linked libraries (Spies *et al.* 2017) were sequenced on the Illumina HiSeq 2500 platform with 150 paired end chemistry. Sequencing generated a total of 713.5 million reads (107 Gbp).

To obtain maximal coverage of expressed genes for transcriptome assembly we collected liver, heart, lung, brain, gonad and salivary gland from the same male and from one female *M. spicilegus*. Tissue was homogenized in Qiazol reagent (Qiagen) with a hand held rotor, and total RNA was extracted using the RNeasy Universal Kit (Qiagen) according to the manufacturer’s instructions. RNA was stored at -80° until processing for Illumina sequencing.

All RNA sequencing libraries were prepared with the Illumina NEBNext Ultra RNA Library Prep Kit according to the manufacturer’s protocol. Libraries were sequenced on the Illumina HiSeq 4000 platform with 150 paired end chemistry. All Illumina generated data for both the genome and transcriptome were quality filtered using standard Illumina recommended quality settings.

### Genome assembly, RNA-seq, genome annotation, and comparative genomics

Genomic linked chromium reads were assembled using the Supernova assembly software version 1.1.5 (Weisenfeld *et al.* 2017). FASTQ linked reads were deconvoluted using the Long Ranger program (10x Genomics) and Illumina’s bcl2fastq software. Supernova was used to assemble the barcoded reads into phased scaffolds. Final scaffolds were produced using Supernova mkoutput pseudohap option. The resulting phased assembly had a size of 2.50 Gbp and was used for all downstream gene calling and subsequent analysis. All generated RNA-seq reads were *de novo* assembled using the RNA assembly program Trinity (Grabherr *et al.* 2011). Run time settings included a minimum contig length of 200bp, 50x coverage read depth normalization, and no use of jaccard clip. All genomic and transcriptome assemblies were conducted on the XSEDE (Towns *et al.* 2014) supercomputer Bridges, operated by the Pittsburg Supercomputing Center.

Peptide sequences were called from the Trinity assembly using the reading frame prediction program Transdecoder (Haas *et al.* 2013). A minimum peptide length of 100 amino acids was used as a length cutoff. Transcript construction, both for final gene model creation and for the training of ab initio gene calling, was accomplished by aligning the assembled Trinity transcripts onto the genome using the transcript alignment program PASA2 (Haas *et al.* 2003), which leverages the EST alignment program GMAP (Wu and Watanabe 2005). PASA alignment was conducted using the gmap alignment software with an average alignment identify of >95% and minimum percent alignment of >75%. Cufflinks (Trapnell *et al.* 2010) based gene models, which were integrated with PASA2, were produced using the standard Cufflinks protocol with a maximum bundle length of 6,500,000. High quality PASA2 transcript models were used to train the gene calling program Augustus (Stanke *et al.* 2006) for exon/intron boundary calling for ab initio predictions. Ab initio models were generated using the trained Augustus species parameters (Hoff and Stanke 2013) with hints provided by Blat transcript alignments (Kent 2002). Blat alignment hints were produced with the following runtime settings: -stepSize = 5 -repMatch = 2253 -minScore = 0 -minIdentity = 0. Protein to genome alignments were created using MouseGRCm38.p5 proteins and NCBI Blast+ (Camacho *et al.* 2009) with the assembled genome. All PASA2 transcript assemblies, protein to genome alignments, and ab initio gene models were combined and consolidated into a single representative model for each gene, using the gene calling program EVidenceModeler (EVM) (Haas *et al.* 2008). Weighted Criteria for gene model construction prioritized transcript to genome alignments (weight = 10) and protein to genome alignments (weight = 10) over ab initio predictions (weight = 5) for the consensus assembly.

All final gene models and predicted transcript peptides were annotated using the Trinotate platform (Bryant *et al.* 2017) with a combination of homology-based search using Blast+, domain identification using hmmscan (Finn *et al.* 2015) and the pfam 30.0 database (Finn *et al.* 2016), and cellular localization with signal P 4.0 (Petersen *et al.* 2011; Hoff and Stanke 2013). In addition, the C-It-Loci (Weirick *et al.* 2015) and Uniprot (Uniprot Consortium 2017) databases were used for functional annotation. Comparative genomic analysis was conducted using a custom Blast database of the predicted proteomes for five *Mus* genomes from the EMBL database (Kulikova *et al.* 2004). E values of 1e-10 or less were considered evidence of homology and were included in a top-hit species based analysis.

Given that the *M. spicilegus* ZRU strain was developed at a facility that also housed wild-derived house mouse strains, we checked for evidence of contamination by searching for chromosomal intervals with few or no SNPs relative to the mouse reference genome. SNP analysis was conducted using bowtie2 (Langmead and Salzberg 2012) alignments against the mouse genome (GRCm38.p5). Quality filters were a minimum quality score of 30 and 25x coverage. Plink (Purcell *et al.* 2007) was used to create a 1MB sliding window with 200kb step intervals.

### Identification and analysis of vomeronasal receptors

We extracted all vomeronasal receptor (VR) sequences from our *M. spicilegus* gene model and transcriptome database and searched for additional VRs in the *M. spicilegus* genome assembly by running a BLASTn homology search against a published dataset comprising VR gene models derived from *M. musculus* vomeronasal transcripts (Dataset S5 from Ibarra-Soria *et al.* 2014). We used the same dataset to annotate VRs in *M. spicilegus*. For *M. spicilegus* sequences with equally high scoring top hits to two or more VRs in the *M. musculus* transcript dataset (*e.g.*, e = 0, % identity within 2%), we ran another BLASTn homology search against the mouse genome. Twenty-six *M. spicilegus* VRs could not be resolved with either approach and are identified by two or more receptor numbers (*e.g.*, *Vmn2r77/78/79*).

Annotated *M. spretus* VR sequences, together with sequences with high homology to a specific VR in the mouse genome, were downloaded using Biomart (Ensembl release 90). All sequences were aligned with MAFFT, implemented in Geneious 10.1.3, and columns with >80% gaps were stripped from the alignment. Inclusion of all known transcript variants from the mouse genome resolved alignment problems caused by incomplete coding sequence for some receptors from *M. spicilegus*. Phylogenetic relationships were inferred using RAxML (Stamatakis 2014) with 100 replicates of rapid bootstrapping. Trees were visualized with the Bioconductor R package ggtree (Yu *et al.* 2017).

### Data availability

Raw data are deposited in the NCBI Sequence Read Archive (Bioproject PRJNA421365; Biosample SAMN08141584; SRA SRP126293). The genome assembly is deposited at DDBJ/ENA/GenBank (accession QGOO00000000), the assembly with original graph information, gene models and transcriptome peptides are available for download at Figshare https://doi.org/10.25387/g3.6137465. Vomeronasal receptor sequences and phylograms are available at FigShare as File S1 and Figure S1 (V1Rs), and File S2 and Figure S2 (V2Rs). Supplemental material available at Figshare: https://doi.org/10.25387/g3.6137465.

## Results

### Overview of read cloud sequencing and genome assembly

The genome of *M. spicilegus* was sequenced using 10x Genomics linked read sequencing (Spies *et al.* 2017). For gene calling and genome annotation we sequenced pooled RNA from multiple tissue types and generated high quality transcripts suitable for gene construction and annotation. Totals of 116 Gbp of genomic read data (56.6x coverage) and 59.6 Gbp of transcriptome data were generated (Table 1).

**Table 1:** *Mus spicilegus* genome and transcriptome raw read and base counts

Value	10x Genome	Transcriptome
Read Pairs	1 550 168 820	19 878 467
Total Bases	116 262 661 500	59 63 540 100

We produced a high quality genome of 2.50 Gbp with a scaffold N50 of 2.27 Mbp. Comprehensive summary statistics are provided in Table 2. Almost all scaffold and contig data were non-ambiguous nucleotides (95.76%). The assembly was highly continuous: more than 90% of the scaffolds were larger than 235 kb. Gene models were created with a combination of Trinity (Grabherr *et al.* 2011) transcript alignments, ab initio trained Augustus models (Stanke *et al.* 2006; Hoff and Stanke 2013), and protein to genome alignments. Combining these sources of evidence produced a total of 28 624 raw gene models (Table 2). Genome completeness was calculated at 99.57%, based on the presence of complete or partial homologs for 232 of 233 conserved single copy genes found using the gVolante (Nishimura *et al.* 2017) webserver with the Busco v3 algorithm (Simão *et al.* 2015). Because our transcriptome data comprised pooled tissues from both sexes, Y-linked genes were not well represented and only one was among our high confidence gene models (*Rbmy*, 98% amino acid identity to mouse NCBI reference). However, cursory Blast searches against our genome with Y gene sequences from the mouse reference genome identified ∼180 kb that included sequences from several single copy genes from the short arm of the mouse Y chromosome (*e.g.*, *Kdm5d*, *Ube1y*, *Uty*, *Zfy2*). Taken together these results support the use of read cloud sequencing to produce very high quality mammalian genomes.

**Table 2:** Genome, transcriptome, and annotation statistics for *M. spicilegus*

*De novo* genome assembly	Value*^a^*
Scaffold N50	2 198 966
Scaffold N90	235 414
Assembly size scaffolds	2 496 544 896
Contig N50	30 918
Contig N90	7729
Contig assembly size	2 390 795 516
Scaffolds 10kb+ N50	2 265 242
Scaffolds 10kb+ N90	413 257
Size 10kb+ scaffolds	2 396 298 463
*De novo* transcriptome	
Number of assembled transcripts	169 733
Total bases in assembled transcriptome	229 968 259
Transcriptome N50	2178
Transcriptome N90	536
Number of predicted proteins	112 521
Number of full length predicted proteins	55 149
Annotated genome	
Number of transcript to genome alignments (GMAP)	771 752
Number of PASA2 assemblies	83 465
Number of AUGUSTUS ab initio models	28 885
Number of protein to genome alignments	16 665
Number of EVM gene models	28 624
Number of final gene models with PASA	26 074
Average gene length	18 265
Average protein length*^b^*	465.2
Average cDNA length	2476.2
Number of exons	334 559
Average number of exons/gene	12.8
Number of genes with Blast hit ≤1e-10	25 557

a Values are reported in base pairs or *^b^*amino acids.

### Gene model comparisons to congeners

To determine homology between our *M. spicilegus* gene models and previously sequenced genomes, we compared our final protein gene models to all the proteins present in the Uniprot Trembl database (Uniprot Consortium 2017) using BLASTp. To reduce redundancy of isoforms we clustered our protein set at 98% similarity using CD-HIT (Huang *et al.* 2010). Using an e-value cutoff of 1e-10 or less, 25 557 of these non-redundant proteins returned a positive hit in the Uniprot Trembl database (Table 3). To evaluate homology relative to other *Mus* species, we ran one to one comparisons between the 25 557 *M. spicilegus* proteins and the genomes of wild-derived strains that represent the closest relatives of *M. spicilegus*: *M. spretus*, the three house mouse subspecies and *M. caroli*, a species that is outside the clade containing house mice, *M. spicilegus* and *M. spretus*. We also included the mouse reference genome, which is a mosaic of all three house mouse subspecies with the largest contribution from *M. m. domesticus* (Yang *et al.* 2011). Using the 1e-10 cutoff, we found a similarly high percentage of positive hits for all comparisons (range, 97.8–96.8%, Table 3).

**Table 3:** Blastp comparison of *M. spicilegus* gene models to other *Mus* species, and the largely *M. m. domesticus*-derived mouse reference genome

Species or strain	Genome*^a^* or database	Positive hits
*Mus spretus*	SPRET_EiJ_v1	24 779
*Mus musculus domesticus*	WSB_EiJ_v1	24 729
C57BL/6J	GRCm38.p5	25 006
*Mus musculus castaneus*	CAST_EiJ_v1	24 771
*Mus musculus musculus*	PWK_PhJ_v1	24 742
*Mus caroli*	CAROLI_EiJ_v1.1	24 768
	Uniprot Trembl	25 557

a Ensembl assembly name.

To infer genomic relationships between *M. spicilegus* and related species, we ran a homology search against a single database containing the five wild-derived *Mus* genomes listed in Table 3. Based on the single best scoring hit for each *M. spicilegus* protein model, the genome with the largest number of top hits was that of *M. spretus* (11 800; Table 4). When top hits to each of the *M. musculus* subspecies’ genomes were combined, homology to *M. musculus* remained slightly lower (11 029; Table 4). Interestingly, within *M. musculus*, there were approximately twice as many top hits to the *M. m. domesticus* genome as to either the *M. m. musculus* or the *M. m. castaneus* genomes (Table 4).

**Table 4:** Blastp homology table for *M. spicilegus* top hits to *Mus* species database

Species	Number of Hits
*M. spretus*	11 800
*M. musculus* combined	11 029
*M. m. domesticus*	5581
*M. m. castaneus*	2606
*M. m. musculus*	2842
*M. caroli*	2147
Total	24 976

Sliding window analysis based on ∼17 million quality-filtered SNPs did not provide any evidence for recent contamination from a *M. musculus* strain. There were no intervals devoid of SNPs and those with a low number of variants were in gene-poor regions, or on the sex chromosomes where coverage may be lower, or were small (≤300kb). We emphasize, however, that this course-grained comparison with the mouse reference genome does not rule out the more interesting possibility of gene flow between *M. spicilegus* and *M. m. musculus* in nature.

### Vomeronasal receptors in M. spicilegus

To evaluate the utility of this genome for identification and comparative analysis of ecologically important genes, we explored the numerical and molecular diversity of vomeronasal receptors (VRs) in *M. spicilegus*. In mice, these chemoreceptors are narrowly tuned to chemical cues in urine, tears, and other excretions, and are critical modulators of social and reproductive behaviors (Del Punta *et al.* 2002; Stowers *et al.* 2002; Haga *et al.* 2010; Doyle *et al.* 2016). VRs comprise three gene families, the numerically diverse V1Rs and V2Rs, and eight formyl peptide receptors (FPRs). Of the >500 VRs annotated in the lab mouse genome, more than 400 are expressed (Ibarra-Soria *et al.* 2014). Currently, only 80 VRs are annotated in the *M. spretus* genome (ensembl.org/Mus_spretus/ accessed November 16, 2017). However, targeted analysis of VR repertoires in a suite of house mouse genomes determined that >80% of VRs in house mice have one to one orthologs in *M. spretus*, with an additional six having evidence of independent duplication in *M. spretus* (Wynn *et al.* 2012). This suggests that the diversity of VRs in house mice should be comparable in aboriginal close relatives, including *M. spicilegus*.

Vomeronasal receptors in *M. spicilegus* were identified and annotated using homology searches against a published VR transcript gene model dataset (Ibarra-Soria *et al.* 2014) and against the mouse reference genome (*M. musculus*; GRCm38.p5). After removing duplicates and VRs annotated as pseudogenes, these approaches recovered a total of 196 high confidence VRs, of which 120 belong to the single exon V1R family (File S1), and 76 belong to the multi-exon V2R family (File S2). We extracted exonic sequences for each family, aligned these to orthologs in the mouse genome, together with available orthologs from the *M. spretus* genome, and estimated phylogenetic relationships using maximum likelihood criteria.

**Figure 2:**
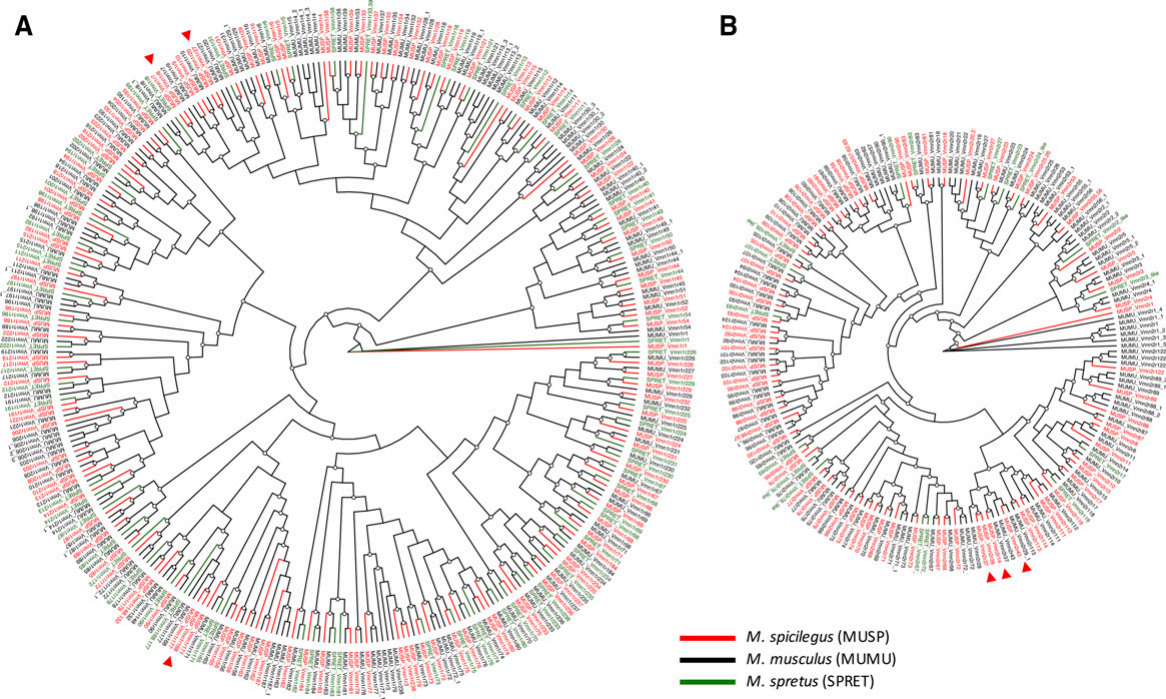
Phylogenetic relationships among the two major vomeronasal receptor subfamilies, V1Rs (A) and V2Rs (B) in *M. spicilegus* (MUSP, red branches and gene names), *M. musculus* (MUMU, black branches and gene names), and *M. spretus* (SPRET, green branches and gene names). Trees are unrooted cladograms, open circles on nodes indicate bootstrap support >90. Red arrowheads indicate *M. spicilegus* receptors that are not sister to orthologs with the same name in either *M. musculus* or *M. spretus*. Gene names with an underscore and number appended are transcript variants in *M. musculus*. Gene names with “like” appended are unannotated putative VRs in the *M. spretus* genome. *M. spicilegus* VR sequences are provided in Supplemental Material (V1Rs: File S1; V2Rs: File S2).

In the majority of cases, each *M. spicilegus* VR was sister to either the *M. musculus* or the *M. spretus* ortholog, or directly basal to both (Figure 2; Figures S1 and S2). For the 60 V1Rs with sequences for all three species, we inferred sister relationships between *M. spicilegus* and *M. musculus*, *M. spicilegus* and *M. spretus*, and *M. musculus* and *M. spretus* for 24, 15 and 21 receptors, respectively (Figure 2a; Figure S1). These values were not significantly different from random expectations (Chi-square = 3.15, *P* = 0.2). The distribution of sister relationships was similarly random for the 12 V2Rs represented by all three species (Figure 2b; Figure S2; *M. spicilegus* + *M. musculus n* = 7, *M. spicilegus* + *M. spretus n* = 2, *M. musculus* + *M. spretus n* = 3; Chi-square = 5.25, *P* = 0.07). More notably, four V2R (*Vmn2r14*, *Vmn2r28*, *Vmn2r37*, *Vmn2r43*) and six V1R (*Vmn1r7*, *Vmn1r8*, *Vmn1r20*, *Vmn1r27*, *Vmn1r168*, *Vmn1r177*) sequences from *M. spicilegus* were more closely related to each other, or to different VRs in *M. musculus*, than they were to the orthologous VRs in *M. musculus* (arrow heads in Figure 2). These receptors are strong candidates for species-specific response to socially relevant chemosignals in *M. spicilegus*.

## Discussion

Using read cloud sequencing, we produced a high quality reference assembly for *Mus spicilegus*, a close relative of house mice that is ecologically and behaviorally unique. Using a single DNA library for a high quality assembly represents a cost and labor efficient method for generating assembly data for mammalian species, and facilitates the production of a large number of genome assemblies for comparative or population genomics. Preliminary comparative analyses of 25 557 protein gene models suggest a slightly closer relationship between *M. spicilegus* and *M. spretus* than between *M. spicilegus* and house mice (represented in our analysis by genomes from the three main subspecies). This inference is consistent with phylogenetic analyses based on whole exome sequencing, which place *M. spicilegus* and *M. spretus* in the sister clade to house mice with *M. spretus* as the basal member of that clade (Sarver *et al.* 2017). Of the three house mouse subspecies, *M. m. domesticus* had the highest homology to *M. spicilegus*. This is also consistent with current phylogenetic hypotheses for the house mouse clade that place *M. m. domesticus* as the basal member (White *et al.* 2009; Keane *et al.* 2011). We note two caveats to this result. First, the genomes of the wild-derived inbred strains used to represent *M. m. castaneus* (CAST/EiJ) and *M. m. musculus* (PWK/PhJ) have low levels of contamination from *M. m. domesticus*-derived classical inbred strains (8% in CAST/EiJ, 6% in PWK/PhJ; Yang *et al.* 2011). This would not, however, bias our inference that *M. spicilegus* peptide sequences share higher homology with *M. m. domesticus* relative to the two other house mouse subspecies. Second, use of the largely *M. m. domesticus*–derived lab mouse genome as a reference for the wild-derived subspecies’ genomes (Kolmogorov *et al.* 2016) might upwardly bias the probability of detecting higher homology to *M. m. domesticus* (*i.e.*, WSB/EiJ) relative to the other two subspecies. We anticipate that the *M. spicilegus* genome will promote additional phylogenetic hypothesis testing that will help to resolve evolutionary relationships between house mice and related species.

Exploratory characterization of the vomeronasal receptor repertoire in *M. spicilegus* provides a foundation for comparative analysis of the molecular and functional diversity of genes that modulate social and reproductive behavior in mice. Indeed, phylogenetic relationships between VR orthologs in *M. spicilegus*, *M. spretus* and *M. musculus* suggest an intriguing pattern of lineage-specific evolution for small subsets of receptors. More generally, these data illustrate the usefulness of this genome for identification of candidate genes underlying species differences in ecology and behavior.

Finally, house mouse subspecies, *M. m. domesticus* and *M. m. musculus*, hybridize in nature (Payseur *et al.* 2004; Janoušek *et al.* 2012) and historic introgression is documented between *M. m. domesticus* and sympatric congener, *M. spretus* (Song *et al.* 2011; Liu *et al.* 2015). However, the possibility of introgression between *M. m. musculus* and *M. spicilegus* is untested. The two species are sympatric throughout the eastern European and Ukrainian range of *M. spicilegus*, and are syntopic in crop fields during the spring and summer breeding season (Muntyanu 1990; Poteaux *et al.* 2008). Fertile F_1_ females were produced from an experimental cross between *M. spicilegus* and a *M. m. domesticus*-derived lab mouse (Zechner *et al.* 1996). Thus, gene flow between mound-building mice and house mice is a formal possibility that is worthy of future study. A high quality genome for *M. spicilegus* will facilitate robust assignment of ancestry patterns in natural populations.
